# The Epidemiology of Patients' Email Addresses in a French University Hospital: Case-Control Study

**DOI:** 10.2196/13992

**Published:** 2021-02-24

**Authors:** Vincent Looten, Antoine Neuraz, Nicolas Garcelon, Anita Burgun, Gilles Chatellier, Bastien Rance

**Affiliations:** 1 Medical Informatics Department Hôpital Européen Georges-Pompidou Assistance Publique-Hôpitaux de Paris Paris France; 2 UMRS 1138 - Centre de Recherche des Cordeliers Université Paris Descartes, Sorbonne Paris Cité INSERM Paris France; 3 Université Paris Descartes Paris France; 4 Department of Medical Informatics Hôpital Necker-Enfant Malades Assistance Publique des Hôpitaux de Paris Paris France; 5 Institut Imagine Université Paris Descartes Université Paris Descartes-Sorbonne Paris Cité Paris France

**Keywords:** email, data privacy, health communication

## Abstract

**Background:**

Health care professionals are caught between the wish of patients to speed up health-related communication via emails and the need for protecting health information.

**Objective:**

We aimed to analyze the demographic characteristics of patients providing an email, and study the distribution of emails’ domain names.

**Methods:**

We used the information system of the European Hospital Georges Pompidou (HEGP) to identify patients who provided an email address. We used a 1:1 matching strategy to study the demographic characteristics of the patients associated with the presence of an email, and described the characteristics of the emails used (in terms of types of emails—free, business, and personal).

**Results:**

Overall, 4.22% (41,004/971,822) of the total population of patients provided an email address. The year of last contact with the patient is the strongest driver of the presence of an email address (odds ratio [OR] 20.8, 95% CI 18.9-22.9). Patients more likely to provide an email address were treated for chronic conditions and were more likely born between 1950 and 1969 (taking patients born before 1950 as reference [OR 1.60, 95% CI 1.54-1.67], and compared to those born after 1990 [OR 0.56, 95% CI 0.53-0.59]). Of the 41,004 email addresses collected, 37,779 were associated with known email providers, 31,005 email addresses were associated with Google, Microsoft, Orange, and Yahoo!, 2878 with business emails addresses, and 347 email addresses with personalized domain names.

**Conclusions:**

Emails have been collected only recently in our institution. The importance of the year of last contact probably reflects this recent change in contact information collection policy. The demographic characteristics and especially the age distribution are likely the result of a population bias in the hospital: patients providing email are more likely to be treated for chronic diseases. A risk analysis of the use of email revealed several situations that could constitute a breach of privacy that is both likely and with major consequences. Patients treated for chronic diseases are more likely to provide an email address, and are also more at risk in case of privacy breach. Several common situations could expose their private information. We recommend a very restrictive use of the emails for health communication.

## Introduction

### Background

In the digital era, there is a natural worldwide trend for patients and physicians to use modern communication tools including emails, texting, social networks, and instant messaging services. Several studies illustrate this trend throughout the world [1-4]. The arrival of *digital natives* [5] among both the health care workforce and the adult population, alongside the development of mobile apps and wearable sensors, is probably going to amplify that trend. However, the digital sharing of health information has strong implications in terms of data privacy, data governance, and data security. Focusing on emails only, several questions can be raised: (1) in terms of data privacy, both the Health Insurance Portability and Accountability Act (HIPAA) in the United States and the General Data Protection Regulation (GDPR) in Europe implement rules regarding the use of emails to exchange medical information. Regardless of these regulations, the fact that patients use either personal or business email accounts raises specific questions in itself. (2) In terms of data governance, the use of cloud-based providers or foreign providers depending on foreign jurisdiction raises questions related to the sovereignty of citizens’ data. (3) Finally, many data privacy breaches have shown how considerable the impact of security on the confidentiality of personal data is (eg, the recent Singapore health data breach [6], or the revelations on the misuse of Facebook data [7]).

Regarding data privacy and electronic communication, the American HIPAA Compliance Guide [8] clearly states that sending private medical messages by SMS text messages or email requires a secure platform following the HIPAA safeguards and therefore should not be done using unsecured usual emails. Similarly, in Europe, health care professionals are not supposed to send health-related information using nonsecured platforms [9].

Regardless of those guidelines, patients may use a variety of email services in their everyday life, including personal email servers (hosted directly by the patient or by a mandated third party), public email providers (free services such as Gmail, Hotmail, or associated with an internet provider, eg, Free.fr or Orange in France), and business email providers (ie, emails addresses hosted by or for the patients’ employer).

The use of business or personal email addresses accessed from the workplace is regulated, and could lead to privacy breaches. The US [10-13] and European [14] regulations clearly state that an employer is allowed to monitor their employees’ communications under different conditions and circumstances (eg, if the employee has consented to such a monitoring or if communications are within the ordinary course of business). Therefore, patients providing their health institution with a business email put themselves at risk of privacy violation. The unsecured exchange of medical information through that channel could constitute a breach of doctor–patient confidentiality for the institution.

Data privacy can also be weakened or even shattered by different types of security breaches such as global surveillance programs (eg, the PRISM program [15] revealed by the press), computer hacking, or abuse of data sharing policies.

In this study, we used a common definition for privacy breach: unauthorized collection, use, or disclosure of personal information.

Previous studies have already described the use of email communication in health care. In a 2013 survey, Lee et al [1] showed that 37% of American patients reported having contacted their physicians via email within the last 6 months, and 18% via Facebook. In 2011, a survey across 14 European countries [4] reported that between 18.7% and 36.3% of patients sent or received at least one email from doctors, nurses, or health care organizations. Characteristics of patients using emails seem well-known. However, to the best of our knowledge the risks of privacy breaches associated with the use of different types of email providers are still to be characterized.

### Study Objective

In this article, we assessed the characteristics of email addresses used by patients attending the European Hospital Georges Pompidou (HEGP) in Paris, France. We described the demographic and minimal clinical characteristics of the patients who provided an email address in contrast to those who did not. We examined what types of email services (free, personal, or business providers) were used by patients, identified the main email service providers, and studied the use of business email accounts. Finally, we discussed the potential issues associated with the type of email services.

## Methods

### Overview

In this section, we detail the data collection and wrangling process, the statistical methodology used to analyze the demographic characteristics, and describe the methodology used to explore email characteristics.

### Study Design

The study had a cross-sectional design, and we followed the STROBE recommendation [16] for the reporting.

### Study Population

#### Data Source

Data were extracted from the clinical information system of HEGP, a 700-bed university hospital located in Paris, France, specializing in oncology, cardiovascular disease, and emergency medicine. HEGP has been using a clinical information system since its opening in 2000 [17] and is certified on the 6th level of the Healthcare Information and Management Systems Society (HIMSS) stage [18]. An i2b2 clinical data warehouse [19] fed with data collected in the clinical information system is also deployed.

#### Inclusion Criteria

The study population includes all patients with a hospital stay or an outpatient visit between January 1, 2000, and November 1, 2018, dates on which data were extracted.

#### Data Extraction

In the remainder of the manuscript, we will refer to the part of an email address located after the character “@” as the *domain name* (ie, Local-part@domainname)

We collected the following data from the hospital’s information system: the domain name of the patient’s email address, sex, year of birth, year of the first contact with the hospital (stay or visit), year of last contact with the hospital, and the presence of their first or last names in the domain name of their email addresses. To preserve privacy, an algorithm automatically substituted the domain name with a random string before the extraction when the domain name included the first or last names of the patient.

### Outcomes

#### Comparison of the Population of Patients According to the Presence of an Email Address in the Database

The presence of an email address in the database may depend on the year of first contact with the hospital. In addition, the use of email address and the administrative registration procedure are time dependent. To mitigate this risk of bias, we matched cases (patients with email address) and controls (patients without email address) on the year of first contact with the hospital using a 1:1 ratio.(univariate matching). The quality of the matching was checked (Multimedia Appendix 1). In addition to demographic data, we measured the chronic comorbidities from the ICD-10 (International Classification of Diseases, 10th revision) adaptation of the Charlson Comorbidity Index [20]. We defined the variable “at least one chronic disease” from the chronic comorbidities included in the ICD-10 phenotyping: myocardial infarction, congestive heart failure, peripheral vascular disease, cerebrovascular disease, dementia, chronic pulmonary disease, connective tissue disease, ulcer disease, mild liver disease, moderate/severe liver disease, diabetes mellitus, hemiplegia, moderate/severe renal disease, diabetes mellitus with chronic complications, any tumor, leukemia, lymphoma, metastatic solid tumor, and AIDS. A more detailed description is available in Multimedia Appendix 2.

#### Classification of Email Addresses

We mapped the most frequently used domain names to companies providing the email service (eg, emails ending with gmail.com, hotmail.com, orange.fr, or free.fr are associated with Google, Microsoft, Orange, and Free corporations, respectively). The companies were identified using prior knowledge and *Whois* requests when necessary. We then evaluated in the curated data the frequency of each of the providers.

We defined a business email as an email provided by someone’s employer. To define business emails, we leveraged a list of free email service providers compiled by Svay [21], secondarily enriched with French and European email providers: voila.fr, dbmail.com, icloud.com, gmx.fr, bluewin.ch, dartybox.com, skynet.be, gmx.com and topnet.tn, numericable.fr, orange.fr, orange.com, wanadoo.fr, numericable.fr, outlook.fr, sfr.fr, neuf.fr, club-internet.fr, noos.fr, numericable.com, free.com, bbox.fr, live.fr. We excluded the domain names appearing in this list and then manually reviewed the remaining.

Personal emails were identified during the extraction process by the presence of the first or the last name in the domain name.

### Statistical Analysis and Implementation

Data were expressed as n (%). Characteristics were compared between groups (included versus excluded population, with email address versus without email address) by Mann–Whitney *U* test for numerical data and chi-square test for categorical data. A logistic regression analysis was performed to determine characteristics associated with having an email address on the matched groups. All analysis were performed using R Statistical Software [22]. The source code and resource files (including the updated list of email providers) are available online [23].

### Ethical Considerations

The study was authorized by the HEGP Institutional Review Board on December 21, 2018. The HEGP Institutional Review Board [24] has waived the requirement to obtain informed consent or specific approval for this study.

## Results

### Population

Between 2000 and August of 2018, we collected 41,004 patient email addresses (4.22% of the total population of patients, ie, 41,004/971,822). Of the total number of patients in the database (971,822 patients), we selected a subgroup of 41,004 patients as a control group (ie, patients who did not provide an email address). The characteristics of the population and the control group are described in Table 1. Statistical tests are presented in Multimedia Appendix 3.

**Table 1:** Characteristics of the patients (before and after matching).

Characteristics			Overall population (before matching; N=971,822)		With email (before matching; N=41,004)		Population (after matching; N=82,008)		Excluded population of matching (N=889,814)
Male, n (%)			485,737 (49.98)		19,463 (47.47)		39,957 (48.72)		445,780 (50.10)
Year of birth, median (Q1-Q3)			1960 (1944-1978)		1964 (1952-1978)		1965 (1951-1981)		1960 (1943-1977)
**Year of birth, n (%)**									
	Before 1950	342,676 (35.26)		9308 (22.70)		20,069 (24.47)		322,607 (36.26)	
	1950-1969	282,381 (29.06)		16,115 (39.30)		27,735 (33.82)		254,646 (28.62)	
	1970-1989	281,390 (28.95)		12,655 (30.86)		26,022 (31.73)		255,368 (28.70)	
	1990 and more	65,375 (6.73)		2926 (7.14)		8182 (9.98)		57,193 (6.43)	
Year of the first contact, median (Q1-Q3)			2009 (2004-2014)		2015 (2011-2017)		2015 (2011-2017)		2008 (2003-2013)
**Year of the first contact, n (%)**									
	2005 and before	283,848 (29.21)		3875 (9.45)		7750 (9.45)		276,098 (31.03)	
	2005-2010	229,823 (23.65)		4198 (10.24)		8396 (10.24)		221,427 (24.88)	
	2010 and after	458,151 (47.14)		32,931 (80.31)		65,862 (80.31)		392,289 (44.09)	
Year of last contact, median (Q1-Q3)			2012 (2006-2016)		2018 (2016-2018)		2017 (2014-2018)		2011 (2005-2015)
**Year of last contact, n (%)**									
	2010 and before	283,848 (29.21)		468 (1.14)		7375 (8.99)		414,718 (46.61)	
	2011-2014	229,823 (23.65)		4198 (10.24)		14,109 (17.20)		215,483 (24.22)	
	2015-2018	458,151 (47.14)		35,609 (86.84)		60,459 (73.72)		259,613 (29.18)	
At least one chronic disease, n (%)			8072 (0.83)		5828 (14.21)		8072 (9.84)		0^a^

^a^ICD-10 (International Classification of Diseases, 10th revision) codes were not included in the electronic heath record before 2008.

### Description of the Email Service Providers

The 10 most frequent email providers and their frequency on the data set are summarized in Table 2.

**Table 2:** Prevalence of the 10 most frequent email providers used by HEGP patients.

Email type and provider		Description (N=41,004)	Headquarters localization
Business, n		2878	
Personal, n		347	
**Email provider, n (%)**		37,779	
	Google	11,730 (28.61)	USA
	Microsoft	7802 (19.03)	USA
	Orange	6402 (15.61)	Europe
	Yahoo!	5071 (12.37)	USA
	Free	2427 (5.92)	Europe
	SFR	1170 (2.85)	Europe
	La Poste	815 (1.99)	Europe
	AOL	330 (0.80)	USA
	NOOS	319 (0.78)	Europe
	Bouygues Telecom	250 (0.61)	Europe

### Identification of Business Emails

Starting from 41,004 email addresses, we identified 37,779 email addresses associated with known email providers. We removed 347 emails likely to use personalized domain names (ie, those that included the first or last name of the patient) and identified 2878 domain names likely to be associated with business email addresses.

For privacy reasons, we did not detail the nature of the companies present in the list. However, we can highlight notable findings: around 100 people used email addresses from our own institution. We also found more than 50 email addresses associated with other governmental institutions.

Unsurprisingly, among the most represented companies, businesses geographically located close to the hospital are on top of the list.

### Matching Case–Control: Comparison of the Population of Patients According to the Presence of an Email Address

Table 3 presents the results of the comparison of the groups with and without an email address. After matching, we observed a significant difference in gender, year of birth, and follow-up (*P*<.001). The proportion of patients born between 1950 and 1969 with an email address was higher (39.30 [16,115/41,004] versus 28.34% [11,620/41,004]), whereas we observed the opposite for patients born after 1989 (7.14% [2926/41,004] versus 12.82% [5256/41,004]). Table 4 presents the results of the logistic regressions.

**Table 3:** Case–control comparison of groups with and without emails.

Comparison		Overall population (N=82,008)	Without email address (N=41,004)	With email address (N=41,004)	*P*
Male, n (%)		39,957 (48.72)	20,494 (49.98)	19,463 (47.47)	<.001
Year of birth, median (Q1-Q3)		1965 (1951-1981)	1967 (1950-1984)	1964 (1952-1978)	<.001
**Year of birth, n (%)**					<.001
	Before 1950	20,069 (24.47)	10,761 (26.24)	9308 (22.70)	
	1950-1969	27,735 (33.82)	11,620 (28.34)	16,115 (39.30)	
	1970-1989	26,022 (31.73)	13,367 (32.60)	12,655 (30.86)	
	1990 and after	8182 (9.98)	5256 (12.82)	2926 (7.14)	
Year of last contact, median (Q1-Q3)		2017 (2014-2018)	2016 (2013-2017)	2018 (2016-2018)	<.001
**Year of last contact, n (%)**					<.001
	2010 and before	7375 (8.99)	6907 (16.84)	468 (1.14)	
	2011-2014	14,109 (17.20)	9247 (22.55)	4862 (11.86)	
	2015-2018	60,459 (73.72)	24,850 (60.60)	35,609 (86.84)	
At least one chronic disease, n (%)		8072 (9.84)	2244 (5.47)	5828 (14.21)	<.001

**Table 4:** Regressions (univariate and multivariate analyses) in the matched groups (N=82,008).

Characteristics		Value		Univariate analysis		Multivariate analysis	
				Crude odds ratio (95% CI)		Adjusted odds ratio (95% CI)	
Male, n (%)		39,957 (48.72)		0.90 (0.88-0.93)		0.85 (0.83-0.88)	
**Year of birth, n (%)**							
	Before 1950		20,069 (24.47)		Reference		Reference
	1950-1969		27,735 (33.82)		1.60 (1.55-1.66)		1.60 (1.54-1.67)
	1970-1989		26,022 (31.73)		1.09 (1.05-1.14)		1.17 (1.12-1.22)
	After 1990		8182 (9.98)		0.64 (0.61-0.68)		0.56 (0.53-0.59)
**Year of last contact, n (%)**							
	2010 and before		7375 (8.99)		Reference		Reference
	2011-2014		14,109 (17.20)		7.76 (7.03-8.58)		7.66 (6.93-8.47)
	2015-2018		60,459 (73.72)		21.1 (19.3-23.3)		20.8 (18.9-22.9)
At least one chronic disease, n (%)		8072 (9.84)		2.86 (2.72-3.01)		2.23 (2.11-2.36)	

## Discussion

### Principal Results

Between 2000 and August 2018, out of 971,822 recorded patients, HEGP patients have provided 41,004 email addresses. Patients providing email addresses were mostly born between 1950 and 1989 (28,770/41,004, 70.16%) and had their first contact (ie, first visit) with the hospital after 2010. Among patients who provided an address, the follow-up duration was mostly less than 3 months (11,309/41,004, 27.58%), or more than 60 months (10,847/41,004, 26.45%).

Among all the email addresses collected (N=41,004), 37,779 corresponded to known email providers. Among the top 10 email providers (covering 36,316/37,779, 96.12%, of known domain names), 65.99% (24,933/37,779) were hosted by companies with headquarters located in the USA, versus 30.13% (11,383/37,779) hosted by companies located in Europe; 7.02% (2878/41,004) of the patients provided business email addresses, and 0.85% (347/41,004) used a personalized domain name.

Compared with patients who did not provide an email address, patients who provided one were older. Table 4 shows that patients suffering from at least one chronic disease were also more likely to provide an email address (odds ratio [OR] 2.23, 95% CI 2.11-2.36).

The systematic collection of email addresses in our institution was put in place only recently, explaining the strong effect of the year of last contact (OR 20.8, 95% CI 18.9-22.9).

We counterintuitively observed a lower proportion of email addresses in younger patients. A selection bias in the hospital population is likely to be blamed. Indeed, our hospital is specializing in cardiovascular diseases, oncology, and emergency medicine. Younger patients mostly treated for nonchronic diseases or visiting the emergency department are less likely to provide their address than older patients with a regular follow-up for a chronic condition in specialized departments.

### Comparison With Prior Work

To the best of our knowledge no institution has provided a description of email providers used by their patients. Newhouse et al [4] have described characteristics of patients using email for health care communication. They showed that the proportion of patients using emails increased with the number of health problems reported, the presence of a current long-standing illness or health problem, the presence of undergoing long-term medical treatment, and the number of visits to the doctor in the last 12 months. These observations are in line with our results. However, they reported a proportion of 18.70% of the French population using emails for health care communication (against 4.22% [41,004/971,822] for our hospital). Newhouse et al [4] also reported a more important use of email for male and younger patients. The difference with our results could be explained by the population studied and a bias of declaration. Their results come from a survey in the general population, whereas our results are reflecting a hospital population. Furthermore, they are based on the declaration of emails by the patients themselves at the hospital registration service. Our results are not comparable with the US survey of Lee et al [1], in which no difference between gender was observed; however, the age distribution is comparable to our population. The study population of the Lee et al [1] survey came from retail pharmacy users.

The comparison with related studies should be made carefully as the French and US populations are not strictly comparable; furthermore, the organization of the French and US health systems are substantially different.

### Limitations

This study has some limitations. The first is a selection bias which may limit the generalizability of its findings. This is a cross-sectional study with a large period of inclusion but our population comes from a university hospital in Paris and is not representative of the national population.

This study only reflects the official collection of email addresses. It is possible that health care professionals use direct email communication with their patients outside of the hospital information system. Therefore, the volume of email addresses collected might be underestimated. However, it is likely that the distribution of domain names remains similar.

Another limitation is a bias in the measurement of chronic comorbidities from the ICD-10 according to [20]. Between 2000 and 2007, our information system did not include ICD-10 codes in the electronic heath records in routine care. Nevertheless, this bias is nondifferential after our matching on the first year of contact.

### Perspectives and Ethical Considerations

#### Privacy Breach: Analysis of Risks

**Figure 1:**
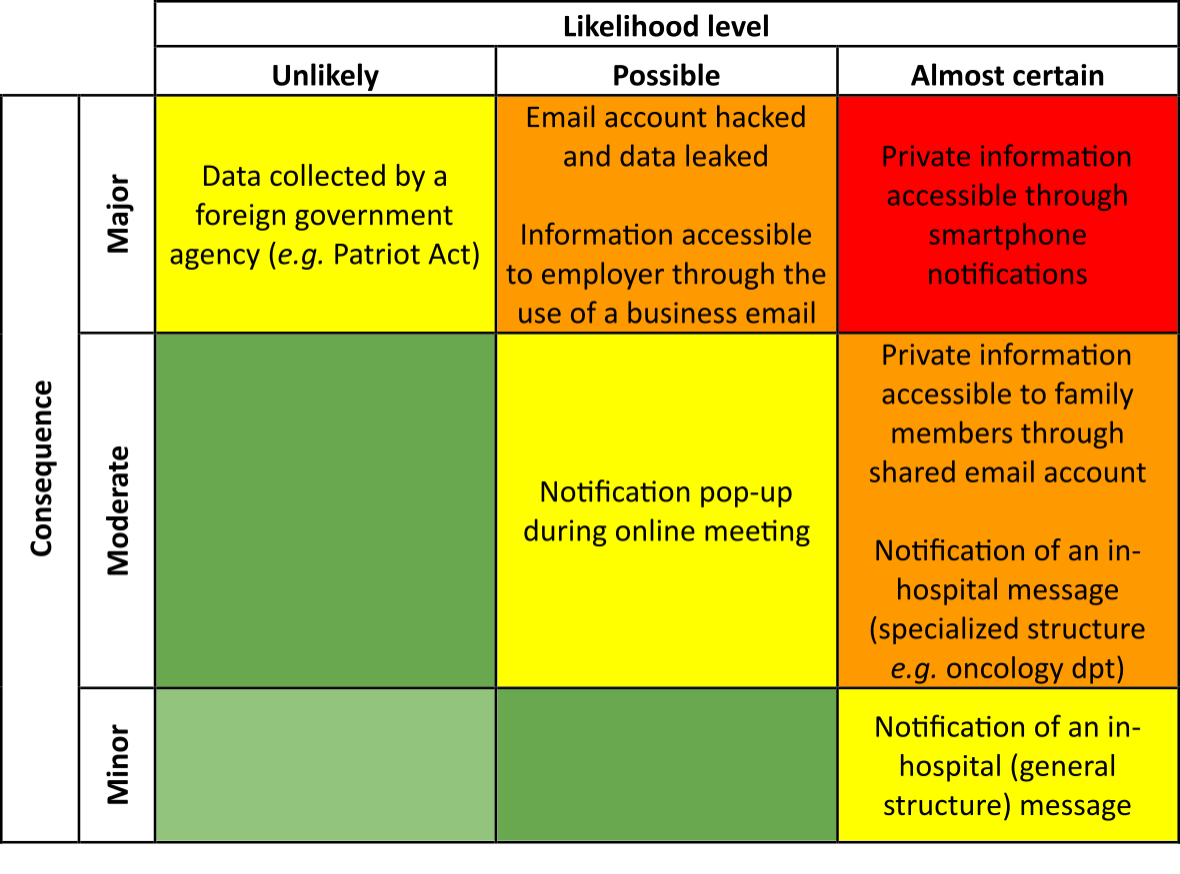
Risk analysis of unauthorized access to private email communication between a hospital and a patient.

Table 4 shows that the patients more likely to have provided the institution with their email address are those suffering from chronic diseases. Social discrimination may be more acute for patient suffering from chronic diseases than for other pathologies. Moreover, these patients are more solicited by hospitals and health care professionals for different reasons: access to results, invitations to participate in clinical studies, and information regarding reuse of the data in retrospective studies. We explored the risks associated with such communication. To determine the risk, we analyzed the likelihood of such an issue and its consequences (Figure 1).

The main risk lies in the interception of the email by a third party. Two main cases appear very probable: (1) breach of privacy through smartphone notifications, given that only a limited number of smartphone users are able to fine-tune the privacy settings of their smartphones and tablets; (2) breach of privacy through family members: the email address provided in the information system may be used by other members of the family. It could be a shared email address with their spouse, the address of a child for elderly patients, or that of a parent for younger patients. The shared address collected at a point in time might not reflect the patients’ aspirations for privacy at a later date.

The hacking of email accounts and information accessed by employers are both likely. It is worth noting that business emails can legally be monitored. In such a case the infrastructure to read and capture the information may already be deployed. In the case of email hacking, additional steps are needed.

Many health professionals now use encrypted emails to exchange information with their colleagues. In such cases, the privacy of the exchange is guaranteed by technological means. However, most of the secured email solutions in health care are designed to protect communications among health care professionals, and not with patients. The direct communication via emails may put the patients at risk of a privacy breach.

#### Strategies for Safe Communication

In the communication between health institutions and patients, 3 strategies can be identified: a push strategy (from the institution to the patient, eg, sending messages and reminders), a pull strategy (from the institution to secured third parties, eg, collecting patients’ information from booking platforms), or a bidirectional strategy. The first 2 are already taken into account with the current communication protocols by most of the health care providers. However, unsolicited direct communication from the patients to their physicians can lead to privacy breaches. Health organizations increasingly rely on secured platforms to transmit health documents to their patients (eg, laboratory results, hospital web-based patient portals [25]). However, the communication between the physicians and their patients need to be included in the exchange solutions. There is here a strong need for secured and user-friendly communication tools for direct communication between patients and physicians.

Note that in the context of health care, the name of the institution can reveal sensitive information (eg, an email from the ABC Oncology Center, or the Infection Disease Department at ABC could inform regarding the pathology of the patient). To mitigate this risk, the use of an email address from a general care provider (eg, ABC hospitals) might be preferable.

#### The Self-Privacy Harms

Patients can expose their health data to harm when they use unsecured communication channels (and especially emails). Regulators engaged the responsibility of the institution through the GDPR and HIPAA guidance (in Europe and the United States), but cannot fully protect citizens against their own data sharing behavior.

Protection of data privacy is based on both accountable health care professionals and *engaged* citizen-patients (informed, implicated, and responsible). The education of citizens regarding data privacy and governance should be promoted and encouraged.

The demand, and legitimate need, for easier ways of communication might overlap strongly with the privacy paradox. First mentioned by Brown in 2001 [26] and defined by Norberg et al [27] in 2007, the privacy paradox is “the relationship between individuals’ intentions to disclose personal information and their actual personal information disclosure behaviors.” It reveals an inconsistency of privacy attitudes and privacy behavior [28]. The use of cash-back rewards cards or free internet services represent famous examples of the privacy paradox. In both cases, the monetary benefits for the user are often inferior to the price the user would assign to the data provided in exchange. In the case of free email providers, virtually all providers are bound by the law to respect and enforce privacy protection.

## Appendix

Multimedia Appendix 1 Quality check of the case-control matching of groups with and without emails.

Multimedia Appendix 2 Chronic disease comparison of case-control groups.

Multimedia Appendix 3 Comparison between the population after matching and the excluded population.

## Abbreviations

GDPR: General Data Protection Regulation
HEGP: European Hospital Georges Pompidou
HIMSS: Healthcare Information and Management Systems Society
HIPAA: Health Insurance Portability and Accountability Act
ICD-10: International Classification of Diseases, 10th revision
OR: odds ratio

## References

[1] Lee JL, Choudhry NK, Wu AW, Matlin OS, Brennan TA, Shrank WH (2016). Patient Use of Email, Facebook, and Physician Websites to Communicate with Physicians: A National Online Survey of Retail Pharmacy Users. J Gen Intern Med.

[2] Brooks RG, Menachemi N (2006). Physicians' use of email with patients: factors influencing electronic communication and adherence to best practices. J Med Internet Res.

[3] Singh H, Fox SA, Petersen NJ, Shethia A, Street RL (2009). Older patients' enthusiasm to use electronic mail to communicate with their physicians: cross-sectional survey. J Med Internet Res.

[4] Newhouse N, Lupiáñez-Villanueva F, Codagnone C, Atherton H (2015). Patient use of email for health care communication purposes across 14 European countries: an analysis of users according to demographic and health-related factors. J Med Internet Res.

[5] Prensky M (2001). Digital Natives, Digital Immigrants Part 1. On the Horizon.

[6] Baynes C (2018). Hackers steal 1.5 million people's personal data in cyber attack on Singapore's health service.

[7] Confessore N (2018). Cambridge Analytica and Facebook: The Scandal and the Fallout So Far. New York Times.

[8] Alder S (2017). HIPAA Compliance Guide. HIPAA Journal.

[9] (2016). Regulation (EU) 2016/679 of the European Parliament and of the Council of 27 April 2016 on the protection of natural persons with regard to the processing of personal data and on the free movement of such data, and repealing. Directive 95/46/EC.

[10] (1979). United States v. Axselle, 604 F.2d 1330, 1338 (10th Cir. 1979).

[11] (1987). United States v. Amen, 831 F.2d 373, 378 (2d Cir. 1987).

[12] (2000). Arias v. Mutual Central Alarm Service, Inc., 202 F.3d 553, 557 n.3 (2d Cir. 2000).

[13] (1993). United States v. Mullins, supra note 16, 992 F.2d,1472 (1993).

[14] Grand Chamber judgment of 12 January 2016 in the case of Barbulescu v. Romania (no. 61496/08). European Court of Human Rights.

[15] Gellman B, Poitras L U. S.

[16] von EE, Altman DG, Egger M, Pocock SJ, Gøtzsche PC, Vandenbroucke JP (2007). The Strengthening the Reporting of Observational Studies in Epidemiology (STROBE) statement: guidelines for reporting observational studies. Lancet.

[17] Degoulet P, Marin L, Lavril M, Le Bozec C, Delbecke E, Meaux J, Rose L (2003). The HEGP component-based clinical information system. Int J Med Inform.

[18] http://www.himssanalytics.org/amam.

[19] Zapletal E, Rodon N, Grabar N, Degoulet P (2010). Methodology of integration of a clinical data warehouse with a clinical information system: the HEGP case. Stud Health Technol Inform Internet.

[20] Sundararajan V, Henderson T, Perry C, Muggivan A, Quan H, Ghali WA (2004). New ICD-10 version of the Charlson comorbidity index predicted in-hospital mortality. Journal of Clinical Epidemiology.

[21] free-email-providers Internet. Svay M.

[22] R Core Team (2015). R: A Language and Environment for Statistical Computing.

[23] Looten Vincent, Rance Bastien EmailsPrivacy.

[24] Jannot A, Zapletal E, Avillach P, Mamzer M, Burgun A, Degoulet P (2017). The Georges Pompidou University Hospital Clinical Data Warehouse: A 8-years follow-up experience. International Journal of Medical Informatics.

[25] Jahn M, Porter B, Patel H, Zillich A, Simon S, Russ A (2018). Usability Assessment of Secure Messaging for Clinical Document Sharing between Health Care Providers and Patients. Appl Clin Inform.

[26] Brown B (2001). Studying the internet experience. HP Laboratories Technical Report (HPL--49) Internet.

[27] Norberg P, Horne D, Horne D (2007). The Privacy Paradox: Personal Information Disclosure Intentions versus Behaviors. J Consum Aff Internet 2007 Jun;.

[28] Kokolakis S (2017). Privacy attitudes and privacy behaviour: A review of current research on the privacy paradox phenomenon. Computers & Security.

