# Supplementary material for: The Epidemiology of Patients' Email Addresses in a French University Hospital: Case-Control Study
Source: J Med Internet Res. 2021 Feb 24;23(2):e13992. doi: 10.2196/13992 (PMC7946586; doi:10.2196/13992)
Supplement: Multimedia Appendix 1 [file jmir_v23i2e13992_app1.docx]

|  | **Population total** | **Without email address** | **With email address** | ***P*** |
| --- | --- | --- | --- | --- |
| **N** | 82,008 | 41,004 | 41,004 |  |
|  |  |  |  |  |
| **Year of the first contact, n(%)** |  |  |  |  |
| 2005 and less | 7,750  (9.5%) | 3,875  (9.5%) | 3,875  (9.5%) | 1 |
| 2005-2010 | 8,396  (10.2%) | 4,198  (10.2%) | 4,198  (10.2%) |  |
| 2010 and more | 65,862  (80.3%) | 32,931 (80.3%) | 32,931  (80.3%) |  |
