# Supplementary material for: The Epidemiology of Patients' Email Addresses in a French University Hospital: Case-Control Study
Source: J Med Internet Res. 2021 Feb 24;23(2):e13992. doi: 10.2196/13992 (PMC7946586; doi:10.2196/13992)
Supplement: Multimedia Appendix 2 [file jmir_v23i2e13992_app2.docx]

|  | **Population total** | **Without email address** | **With email address** | ***P*** |
| --- | --- | --- | --- | --- |
| **N** | 82,008 | 41,004 | 41,004 |  |
|  |  |  |  |  |
| Myocardial infarction | 555 (0.68%) | 205 (0.50%) | 350 (0.85%) | <0.001 |
| Congestive heart failure | 2,427 (2.96%) | 841 (2.05%) | 1,586 (3.87%) | <0.001 |
| Peripheral vascular disease | 3,981 (4.85%) | 1,051 (2.56%) | 2,930 (7.15%) | <0.001 |
| Cerebrovascular disease | 1,790 (2.18%) | 576 (1.40%) | 1,214 (2.96%) | <0.001 |
| Dementia | 366 (0.45%) | 254 (0.62%) | 112 (0.27%) | <0.001 |
| Chronic pulmonary disease | 2,278 (2.78%) | 699 (1.70%) | 1,579 (3.85%) | <0.001 |
| Connective tissue disease | 583 (0.71%) | 168 (0.41%) | 415 (1.01%) | <0.001 |
| Ulcer disease | 258 (0.31%) | 56 (0.14%) | 202 (0.49%) | <0.001 |
| Mild liver disease | 528 (0.64%) | 97 (0.24%) | 431 (1.05%) | <0.001 |
| Moderate/severe liver disease | 279 (0.34%) | 83 (0.20%) | 196 (0.48%) | <0.001 |
| Diabetes mellitus | 1920 (2.34%) | 481 (1.17%) | 1439 (3.51%) | <0.001 |
| Hemiplegia | 199 (0.24%) | 88 (0.21%) | 111 (0.27%) | 0.12 |
| Moderate/severe renal disease | 3878 (4.73%) | 1228 (2.99%) | 2650 (6.46%) | <0.001 |
| Diabetes mellitus with chronic complications | 1490 (1.82%) | 435 (1.06%) | 1055 (2.57%) | <0.001 |
| Any tumor | 7343 (8.95%) | 1581 (3.86%) | 5762 (14.1%) | <0.001 |
| Leukemia | 133 (0.16%) | 47 (0.11%) | 86 (0.21%) | 0.001 |
| Lymphoma | 298 (0.36%) | 107 (0.26%) | 191 (0.47%) | <0.001 |
| Metastatic solid tumor | 2352 (2.87%) | 524 (1.28%) | 1828 (4.46%) | <0.001 |
| AIDS | 574 (0.70%) | 46 (0.11%) | 528 (1.29%) | <0.001 |
