# Supplementary material for: The Epidemiology of Patients' Email Addresses in a French University Hospital: Case-Control Study
Source: J Med Internet Res. 2021 Feb 24;23(2):e13992. doi: 10.2196/13992 (PMC7946586; doi:10.2196/13992)
Supplement: Multimedia Appendix 3 [file jmir_v23i2e13992_app3.docx]

|  | **Population**  **(after matching)** | **Excluded population**  **of matching** | ***P*** |
| --- | --- | --- | --- |
| **N** | 82,008 | 889,814 |  |
|  |  |  |  |
| Male, n (%) | 39,957  (48.7%) | 445,780 (50.1%) | <0.001 |
| **Year of birth, median (Q1-Q3)** | 1965  (1951-1981) | 1960  (1943-1977) | <0.001 |
| **Year of birth, n (%)** |  |  |  |
| Before 1950 | 20,069  (24.5%) | 322,607 (36.3%) | <0.001 |
| 1950-1969 | 27,735  (33.8%) | 254,646 (28.6%) |  |
| 1970-1989 | 26,022  (31.7%) | 255,368 (28.7%) |  |
| 1990 and more | 8,182  (10%) | 57,193 (6.4%) |  |
| **Year of the first contact, median (Q1-Q3)** | 2015  (2011-2017) | 2008  (2003-2013) | <0.001 |
| **Year of the first contact, n(%)** | 2015  (2011-2017) | 2008  (2003-2013) | <0.001 |
| 2005 and less | 7,750  (9.5%) | 276,098 (31.0%) | <0.001 |
| 2005-2010 | 8,396  (10.2%) | 221,427 (24.9%) |  |
| 2010 and more | 65,862  (80.3%) | 392,289 (44.1%) |  |
| **Year of last contact, median (Q1-Q3)** | 2017  (2014-2018) | 2011  (2005-2015) | <0.001 |
| **Year of last contact, n (%)** |  |  |  |
| 2010 and before | 7,375  (9%) | 414,718 (46.6%) | <0.001 |
| 2011-2014 | 14,109  (17.2%) | 215,483 (24.2%) |  |
| 2015-2018 | 60,459  (73.8%) | 259,613 (29.2%) |  |
| **At least one chronic disease, n(%)** | 8,072  (9.8%) | 0* | <0.001 |
